# Guided Internet-based versus face-to-face clinical care in the management of tinnitus: study protocol for a multi-centre randomised controlled trial

**DOI:** 10.1186/s13063-017-1931-6

**Published:** 2017-04-21

**Authors:** Eldré W. Beukes, David M. Baguley, Peter M. Allen, Vinaya Manchaiah, Gerhard Andersson

**Affiliations:** 1 Department of Vision and Hearing Sciences, Anglia Ruskin University, Cambridge, UK; 2 National Institute for Health Research [NIHR], Nottingham Biomedical Research Centre, Ropewalk House, 113 The Ropewalk, Nottingham, UK; 3 Otology and Hearing Group, Division of Clinical Neuroscience, School of Medicine, University of Nottingham, Nottingham, UK; 4 Vision and Eye Research Unit, Anglia Ruskin University, Cambridge, UK; 5 Department of Speech and Hearing Sciences, Lamar University, Beaumont, Texas USA; 6 Linnaeus Centre HEAD, Swedish Institute for Disability Research, Department of Behavioural Science and Learning, Linköping University, Linköping, Sweden; 7 Audiology India, Mysore, Karnataka India; 8 Department of Speech and Hearing, School of Allied Health Sciences, Manipal University, Manipal, Karnataka India; 9 Department of Behavioural Sciences and Learning, Linköping University, Linköping, Sweden; 10 Division of Psychiatry, Department of Clinical Neuroscience, Karolinska Institute, Stockholm, Sweden

**Keywords:** Service development, Tinnitus management, Clinical intervention, Tinnitus distress, Non-inferiority trial, Tinnitus treatment, Internet intervention, Cognitive behavioural therapy, Guided intervention

## Abstract

**Background:**

Innovative strategies are required to improve access to evidence-based tinnitus interventions. A guided Internet-based cognitive behavioural therapy (iCBT) intervention for tinnitus was therefore developed for a U.K. population. Initial clinical trials indicated efficacy of iCBT at reducing tinnitus severity and associated comorbidities such as insomnia and depression. The aim of this phase III randomised controlled trial is to compare this new iCBT intervention with an established intervention, namely face-to-face clinical care for tinnitus.

**Methods/design:**

This will be a multi-centre study undertaken across three hospitals in the East of England. The design is a randomised, two-arm, parallel-group, non-inferiority trial with a 2-month follow-up. The experimental group will receive the guided iCBT intervention, whereas the active control group will receive the usual face-to-face clinical care. An independent researcher will randomly assign participants, using a computer-generated randomisation schedule, after stratification for tinnitus severity. There will be 46 participants in each group. The primary assessment measure will be the Tinnitus Functional Index. Data analysis will establish whether non-inferiority is achieved using a pre-defined non-inferiority margin.

**Discussion:**

This protocol outlines phase III of a clinical trial comparing a new iCBT with established face-to-face care for tinnitus. If guided iCBT for tinnitus proves to be as effective as the usual tinnitus care, it may be a viable additional management route for individuals with tinnitus. This could increase access to evidence-based effective tinnitus care and reduce the pressures on existing health care systems.

**Trial registration:**

ClinicalTrials.gov identifier: NCT02665975. Registered on 22 January 2016.

**Electronic supplementary material:**

The online version of this article (doi:10.1186/s13063-017-1931-6) contains supplementary material, which is available to authorized users.

## Background

Tinnitus is a complex phenomenon characterised by sounds that are consciously perceived in the absence of an external sound source [1]. Owing to the heterogeneity of tinnitus, pharmacological and medical treatments are often unsuccessful, and a cure is still being sought [2]. Experiencing tinnitus may negatively affect many aspects of daily life, including sleep, mood and concentration [3]. It can therefore be debilitating and reduce quality of life. Attending specialised tinnitus clinics may significantly reduce functional and social disability related to tinnitus. Audiological professionals frequently provide this care. They play a major role in offering support to patients experiencing tinnitus. This is largely due to the relationship between tinnitus and hearing loss, although tinnitus can occur without hearing loss [4]. Tinnitus clinics in the United Kingdom are encouraged to provide tinnitus management in line with good practice guidelines [5]. Recommended management approaches include informational counselling, patient education, sound therapy, relaxation therapy, sleep management, the fitting of hearing aids or wearable sound generators, and the use of cognitive behavioural therapy (CBT). Audiology departments in the United Kingdom vary regarding which of these services they offer and often use a combination of approaches [6]. Research supporting the effectiveness of many of these interventions in isolation is limited or hampered by poor methodologies. There is, however, a wide range of research supporting the efficacy of CBT in reducing tinnitus distress [7, 8]. In addition, CBT has been shown to reduce the effects of a range of conditions, such as insomnia, anxiety, depression and pain [9]. It is a practical solution-focused therapeutic approach aimed at modifying unhelpful thought patterns in order to promote tinnitus habituation [10]. CBT for tinnitus is a comprehensive programme encompassing applied relaxation, cognitive restructuring, and addressing emotional reactions and problems related to having tinnitus [11]. A structured approach is recommended which includes goal setting, active participation and relapse prevention. Of interest is that both efficacy research and patient experience have indicated CBT to be of value as a tinnitus intervention. Informational counselling and CBT were rated more effective than the use of sound therapy and fitting of hearing aids or sound generators by patients attending one audiology department in England [12].

Although tinnitus interventions are of value, they may be extensive, and encompass referrals to various disciplines [13]. The cost of tinnitus to the National Health Service (NHS) in England is estimated to exceed £4.9 million annually to cover only the initial general practitioner (GP) appointment and one outpatient appointment [14]. This does not account for additional assessments, procedures, prescriptions or referrals that are often required. The economic costs of tinnitus are therefore substantial. Further concerns are that not everyone with significant tinnitus has access to these specialist services [6]. Many of the estimated 750,000 people making GP appointments every year in England with tinnitus as the primary complaint are never referred for any specialist tinnitus care [15]. In addition, the global burden of tinnitus appears to be on the increase [14]. A possible reason is a rise in professional and leisure-related noise exposure, which comprise one of the greatest risk factors for developing tinnitus [16]. This increase is likely to place further constraints on health care systems that are already strained. Innovative planning is required to ensure that systems are able to meet these additional pressures. The use of Internet-based cognitive behavioural therapy (iCBT) has been incorporated into regular care in Europe to address these demands [17, 18]. Because no such management route is available in the United Kingdom, an iCBT intervention was developed for those with tinnitus in the United Kingdom [19], using the CBT content published by Kaldo and colleagues [20]. A three-phase clinical trial was designed to evaluate this intervention. One of the aims was to establish whether an audiologist could guide iCBT, in contrast to a clinical psychologist, who delivered previous iCBT trials. The initial pilot study indicated the feasibility and acceptability of this intervention in terms of recruitment, compliance, attrition rates and use of audiological support [21]. Phase II was a delayed treatment efficacy randomised controlled trial [22]. Undertaking the iCBT intervention led to a significant reduction in tinnitus severity (Cohen’s *d* = 0.69) as measured by the Tinnitus Functional Index (TFI) [23]. In addition, a reduction in many of the comorbidities often associated with tinnitus, such as insomnia, depression and hyperacusis, and an improvement in life satisfaction after undertaking the intervention were found. These results remained stable 2 months post-intervention. What is unknown is how outcomes using this new iCBT intervention compare with those of established face-to-face (F2F) clinical care for tinnitus in the United Kingdom. This article describes a study protocol to compare these interventions.

## Methods/design

### Study objectives

The primary aim of this study will be to evaluate whether iCBT for tinnitus is at least as effective as established F2F care in reducing tinnitus severity. The key secondary objective is to compare the effects of these interventions for tinnitus-related comorbidities such as insomnia, depression and anxiety. A further objective is to assess stability of results 2 months post-intervention. Another aim is to establish whether there are any predictor variables associated with outcomes for iCBT compared with those for the usual F2F care. The hypothesis is that iCBT is not inferior to F2F care and that effects will be stable over the follow-up period.

### Study design

**Fig. 1:**
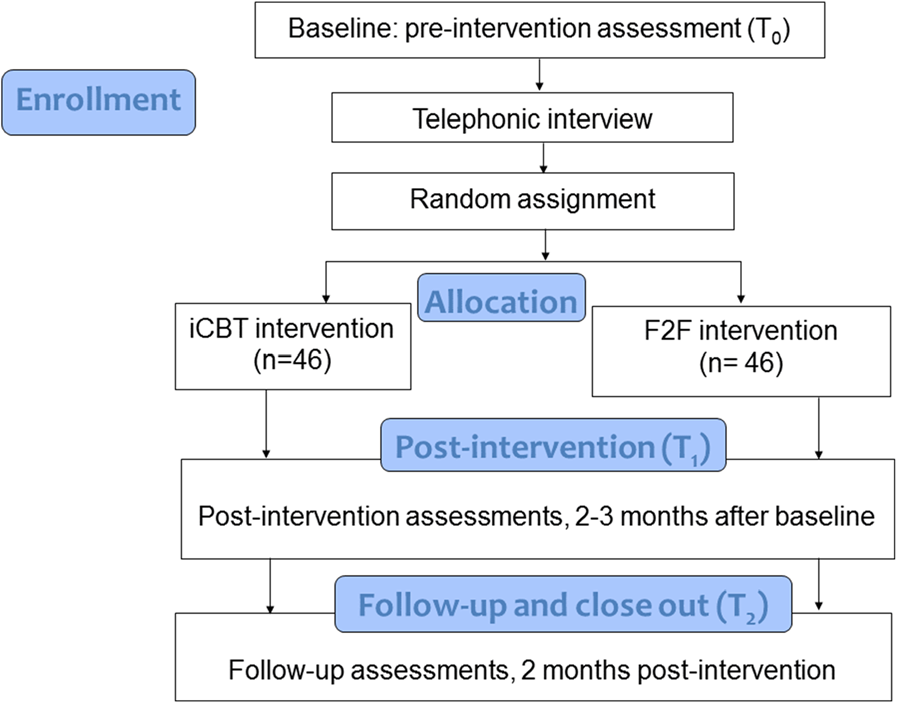
Consolidated Standards of Reporting Trials (CONSORT) study flow diagram. *F2F* Face-to-face, *iCBT* Internet-based cognitive behavioural therapy

A randomised, multi-centre, two-arm, parallel-group, non-inferiority trial with a sequential adaptive design and a 2-month follow-up will be conducted. The experimental intervention, namely guided iCBT, will be compared directly with an active control group, namely usual individual F2F audiological care, as shown in the Consolidated Standards of Reporting Trials (CONSORT) flow diagram [24] in Fig. 1.

This study protocol is described using the Standard Protocol Items: Recommendations for Interventional Trials (SPIRIT) checklist [25, 26], which is included in Additional file 1.

### Study population

#### Recruitment

The investigational sites were selected from among sites partnered in the East of England Tinnitus Network to improve consistency of practise across sites. To increase chances of achieving the target sample size, three England based primary care hospitals were selected, namely Norfolk and Norwich Universities Hospitals Trust, Milton Keynes University Hospital NHS Foundation Trust and Hinchingbrooke Health Care NHS Trust, which all have reputable clinical tinnitus services. The study sponsor and central trial management centre is at Anglia Ruskin University, Cambridge, UK. All adult patients referred to the participating tinnitus clinics during the recruitment period who meet the inclusion criteria will be invited to participate.

#### Inclusion criteria

Participant eligibility for the study is as follows:

1. Aged 18 years or older, living in the United Kingdom and having the ability to read and type in English
2. Regular access to a computer and the Internet and the ability to use these
3. Examined clinically by an ear, nose and throat (ENT) specialist and an audiologist to rule out any medical causes for tinnitus. This evaluation would typically include a case history, otoscopy, tympanometry, hearing test and, where indicated, magnetic resonance imaging.
4. Referred to the tinnitus clinic by an ENT specialist or audiologist because of troublesome tinnitus. Standard protocols will be followed whereby this decision will have been made on the basis of the presenting symptom profile and not on the use of a tinnitus assessment measure.

#### Exclusion criteria

Exclusion criteria are as follows:

1. Reporting any major medical, psychiatric or mental disorder which may hamper commitment to the programme
2. Undergoing any tinnitus therapy concurrently to partaking in this study

### Enrolment and randomisation

Patients who satisfy the eligibility criteria following the screening process will be enrolled and randomised in a 1:1 ratio to either intervention arm by an independent research assistant using a computer-generated randomisation schedule, after stratification for tinnitus severity. Variable, randomly permuted block sizes of 4 and 6 will be used. Whilst a blinded design would be optimal, in this context it is not feasible. Participants allocated to the experimental group will receive the guided iCBT intervention, whereas those in the active control group will receive care at their local hospital. Both participants and the clinicians will therefore know the group allocation. The data analyst will, however, be masked during data analysis. Following allocation, participants will be contacted by the central research team to be informed of the group to which they have been randomised and when their treatment will commence.

### Withdrawal/discontinuation of participants

Strategies to improve adherence to the intervention protocols and minimise attrition rates will be applied as recommended by Dziura and colleagues [27]. These include data collection not requiring clinical appointments and the provision of regular, guided contact during the trial. Participation is voluntary with the right to withdraw without penalty. In rare cases, participants will be withdrawn if, owing to unforeseen circumstances, they are no longer able to participate. Reasons for withdrawal will be recorded.

### Assessment measures

Self-reported assessment measures are used in clinical practice to quantify tinnitus severity and the presence of other psychological conditions. These will therefore be utilised to measure intervention effect, as shown in Table 1. Online questionnaire delivery will be applied consistently throughout the study for both groups. The same questionnaires used in phases I and II of this clinical trial will be utilised to maintain consistency, with the addition of the Tinnitus Handicap Inventory (THI) [28]. A non-validated demographic questionnaire will be used to establish health-related and tinnitus-specific information. Permission has been obtained to use the assessment measures as required.

**Table 1:** Standard Protocol Items: Recommendations for Interventional Trials (SPIRIT) guideline schedule of enrolment, interventions and assessments for both intervention groups

Study period	Enrolment	Intervention	Post-intervention	Follow-up
Measurement time point	T_0_ (baseline)	Lasting 2 months on average	T_1_ (2–3 months after baseline)	T_2_ (2 months post- intervention)
Enrolment				
Informed consent	X			
Online screening questionnaire	X			
Telephone screening	X			
Intervention allocation		X		
Assessments				
Tinnitus Functional Index	X		X	X
Tinnitus Handicap Inventory	X		X	X
Insomnia Severity Index	X		X	X
Patient Health Questionnaire	X		X	X
Generalised Anxiety Disorder	X		X	X
Hyperacusis Questionnaire	X		X	X
Hearing Handicap Inventory	X		X	X
Cognitive Failures Questionnaire	X		X	X
Satisfaction with Life Scale	X		X	X
Tinnitus Handicap Inventory-Screening Version		Weekly for 8 weeks		
Post-intervention telephone call			X	

#### Primary assessment measure

The TFI [23] has been selected as the primary assessment measure because of its validation for assessing intervention responsiveness. The THI [28] will also be administered for comparative purposes because this is the most commonly used assessment measure in clinics in the United Kingdom and is frequently used in clinical trials [29].

#### Secondary assessment measures

Assessment measures related to areas which may be affected by tinnitus have been selected as follows:

1. Sleep difficulties and associated worries about sleep are prevalent amongst those with tinnitus. The Insomnia Severity Index [30] will be included to assess sleep duration, quality of sleep and the impact of sleep habits on well-being.
2. The prevalence of anxiety and depression is high in those with severe tinnitus [31]. To quantify these levels, we will incorporate the Patient Health Questionnaire [32] to measure depression severity and the Generalised Anxiety Disorder [33] to assess anxiety severity.
3. Owing to the large overlap in the prevalence of tinnitus and a reduced tolerance of everyday sounds [34], otherwise known as *hyperacusis*, the Hyperacusis Questionnaire [35] will be administered.
4. Those with distressing tinnitus often find it difficult to focus on other sounds or conversations because of the penetrating nature of their tinnitus. The Hearing Handicap Inventory for Adults-Screening Version [36] will therefore be administered.
5. Tinnitus impairs cognitive function because of its impact on the control of attention [37]. To assess proneness to committing cognitive slips and errors in the completion of everyday tasks, such as failures in perception, memory and motor function, the Cognitive Failures Questionnaire [38] will be included.
6. To include an appropriate measure of the quality of life of those with tinnitus, as opposed to self-care and mobility, the Satisfaction With Life Scale [39] was selected to assess global life satisfaction.

#### Weekly assessment measure

Participants will be monitored weekly using the Tinnitus Handicap Inventory-Screening Version (THI-s) [40], a concise measure consisting of ten questions. During previous trials using this iCBT intervention for tinnitus, researchers found that tinnitus severity decreased on a weekly basis [21]. Once participants reached the fourth week of the intervention, their scores were significantly lower than their baseline scores. It is hypothesised that this will again be the case for the iCBT group in the present study. For those undergoing the F2F intervention, this reduction may occur at an earlier time point, owing to the more intense nature of their initial care.

#### Assessment measurement schedule

All assessment measures will be collected online for both groups using the following measurement schedule:

- *Pre-intervention baseline measurements (T*_*0*_ *):* Baseline measurements will be collected following study registration and prior to allocation.
- *Weekly assessment measurements (during intervention):* Whilst in the intervention phase, both groups will be monitored for an 8-week period by means of the THI-s. Participants from both groups will be in active intervention for an average duration of 2 months, although there may be some individual variation from this. Those allocated to the iCBT experimental group will start the iCBT intervention following allocation. Those in the active F2F control arm will commence hospital-based intervention in the first available clinical opening, typically 1–4 weeks post-allocation.
- *Post-intervention measurements (T*_*1*_ *):* Data will be collected post-intervention, typically 2–3 months following baseline data collection. The same assessment measures administered at baseline will be completed.
- *Follow-up intervention measurements (T*_*2*_ *):* Follow-up data will be collected 2 months post-intervention to determine the stability of intervention effects at this time point.

The specific assessment measures for each collection point are shown in Table 1. To improve attrition rates at follow-up, e-mail reminders will be sent to encourage participants to complete the questionnaires.

#### Semi-structured interviews

Participants who complete the online screening will be contacted telephonically by the central research team. These interviews will be recorded and transcribed for qualitative analysis. They will provide the opportunity to ask about participants’ expectations and motivations. They will also offer the chance to discuss aspects of the study and answer any questions. This initial contact has been found to be valuable in ensuring participants are motivated to complete the programme [41]. After completion of the post-intervention questionnaire, participants will be telephoned again to discuss their progress and find out more about their experiences during participation.

### Study interventions

The following intervention groups will be running in parallel:

- The *experimental iCBT group*, which will receive the iCBT intervention over an 8-week period
- The *F2F active control group*, which will be under the care of their local hospital for an average duration of 8 weeks and attend an average of two or three appointments

#### Intervention outline for both groups

- The estimated duration of active intervention is an 8-week period for both groups, although some individual variation may occur.
- Information about managing tinnitus will be provided to both groups. The delivery of this information will differ, however, being provided online for the iCBT group and F2F for the active F2F control group.
- A log will be kept of the information provided to individuals in both groups. This will be the modules actually done by the iCBT group participants and content covered during appointments for individuals in the F2F group.
- During the initial clinical examination, all participants will be assessed regarding their suitability for hearing aids or combination devices. Where indicated, these will be provided regardless of group allocation.
- An audiologically trained professional will support both groups. This may be a hearing therapist, audiologist or clinical scientist in audiology. Criteria for inclusion of clinicians (providing the intervention to both groups) will be to have had training and experience in managing patients with tinnitus, to be part of a professional tinnitus network and to maintain good clinical practice. In this way, the interventions provided were standardised as much as possible despite participants’ attending different hospitals. The clinicians also agreed to abide by a structured protocol in order for similar components to be received by all participants.

#### Guided iCBT intervention outline (experimental group)

The experimental group will commence the iCBT intervention following group allocation. The CBT content is based on a self-help programme (iCBT) originally developed by Andersson and colleagues [20, 42]. The focus of this intervention is to address the physical, emotional and problematic effects of experiencing tinnitus to aid habituation to tinnitus. Key audiological principles, such as the use of sound enrichment, are also incorporated into the programme. The content of the original programme has been redeveloped for a U.K. population into an interactive e-learning version to ensure that it is visually stimulating, engaging and responsive to participant’s progress [19]. The intervention is partly tailored to individual needs and consists of 16 recommended modules and 5 optional modules, as shown in Table 2. Modules will be released on a weekly basis over an 8-week period. Participants will be instructed to engage with the modules and then practise the suggested techniques on a daily basis. The programme is therefore comprehensive, offering a range of key CBT techniques to maximise behaviour change. The information can be read online, downloaded to be read offline, or printed. The modules contain a mixture of information, videos, quizzes, diagrams, suggested techniques to apply in daily life, worksheets to keep track of progress, and solutions for common problems. There is a secure messaging system to enable participants to ask questions and allow their assigned audiologists to provide feedback.

**Table 2:** Weekly guided Internet-based cognitive behavioural therapy intervention modules for the experimental group

Time line	Intervention content	Intervention load	
		Weekly reading	Daily practising
Week 1	Programme rational and outline	15 minutes	
	Understanding tinnitus	15 minutes	
Week 2	Deep relaxation	10 minutes	10 minutes
	Positive imagery	10 minutes	5 minutes
	Sound enrichment^a^	10 minutes	As required
Week 3	Diaphragmatic breathing	10 minutes	10 minutes
	Reinterpreting tinnitus	10 minutes	5 minutes
	Sleep management^a^	15 minutes	As required
Week 4	Entire body relaxation	10 minutes	5 minutes
	Focussing techniques	10 minutes	5 minutes
	Concentration management^a^	10 minutes	As required
Week 5	Rapid relaxation	10 minutes	3 minutes
	Thought analysis	15 minutes	3 × 15 minutes
	Reducing sound sensitivity^a^	15 minutes	Daily
Week 6	Relaxation in daily routines	10 minutes	3–5 situations
	Cognitive restructuring	15 minutes	3 × 15 minutes
	Communication tactics^a^	15 minutes	As required
Week 7	Relaxation in stressful situations	10 minutes	As required
	Exposure to tinnitus	10 minutes	3 × 5 minutes
Week 8	Reviewing helpful techniques	20 minutes	Evaluation
	Maintenance and relapse prevention	20 minutes	Future plan

^a^Optional modules to be done if required

**Table 3:** Face-to-face intervention content for the control group

Timeline	Intervention content to be individually tailored and may include	Intervention load	
		Explanation	Daily practising
Initial appointment	In-depth case history	20 minutes	
	Information about tinnitus	20 minutes	
	Sound enrichment advice and equipment demonstration	20 minutes	As required
Follow-up appointment	Recap	5 minutes	
	Relaxation advice	15 minutes	10 minutes
	Sleep management advice	20 minutes	As required
	CBT techniques such as identifying negative automatic thoughts	20 minutes	As required
Second follow-up appointment	Review difficulties and address these	20 minutes	As required
	Advice on further support (e.g., tinnitus support groups, charities, tinnitus apps)	20 minutes	As required
	Further options (e.g., mindfulness, hypnosis or concentration management)	20 minutes	As required

*CBT* Cognitive behavioural therapy

#### Face-to-face intervention outline (active control intervention)

The F2F group will receive F2F individualised therapy for tinnitus using the usual informational counselling approach generally followed in the management of tinnitus in the United Kingdom. A structured protocol including similar intervention components was developed to standardise the care received across the different hospitals,  as seen in Table 3. This content will, however, be tailored to each individual. The initial appointment will generally be used to provide explanations about tinnitus and the effects thereof on the individual’s day-to-day life and provide some basic management strategies. A follow-up appointment will be made for 1 month later to discuss additional strategies for tinnitus management. One month later a second follow-up appointment may be made to further address remaining difficulties. These appointments will last 60 minutes, on average, although they may be shorter for those not requiring as much input. The total time under active intervention will be 2 months, on average.

### Safety and clinical monitoring

Protocols to minimise the risks to participants and the researcher have been put in place. The data, together with any other spontaneously reported adverse events during the intervention, will be reported. If any participants are identified as requiring additional support, a letter will be provided for them to take to their GP so that this care can be arranged.

Participants in the iCBT experimental group will be monitored by the audiologist evaluating their worksheets and with communications via a secure online messaging system. This therapeutic alliance will allow for feedback and assistance if participants have any difficulties. Participants in the F2F group will be monitored by the audiology professional they see.

In addition, all participants will be monitored on a weekly basis during the course of the study by means of the THI-s. If a participant’s scores suddenly worsen, the participant will be appropriately managed.

### Data management

The central electronic online data-capturing system is held in Linköping University (Sweden) because of their expertise in Internet interventions. The web portal has appropriate policies and procedures in place complying with the following U.K. legislation: the Data Protection Act [43] and the Privacy and Electronic Communications (EC Directive) Regulations [44]. Appropriate technical and organisational measures have been taken to safeguard the security of the web portal; the servers are located in a locked computer room to which only authorised personnel have access by using cards and keys. It is also not possible to establish a link between the data and individual users through access to the database. Data will be kept on the secure web portal. Data exported for statistical analysis will be kept for 1 year following the end of the study at http://www.data-archive.ac.uk/ and then destroyed following this point.

All personal data will be kept confidential. Each participant will be assigned a random user code (four digits followed by four letters). This will be used by clinicians to identify the participant during the trial. All data communication between servers and users are encrypted (via TLS/https), and all sensitive data will be stored encrypted in the database, using algorithms such as hash message authentication code/secure hash algorithm 256/secret keys.

The researchers, statisticians and internal data monitoring committee will have access to the final dataset. They will ensure accurate analysis and results interpretation. The sponsor of the study will have no role in the study design, data collection, data analysis, data interpretation or writing of the report.

### Data analysis

Data analysis will be performed in accordance with CONSORT guidelines for randomised clinical trials [24, 45]. IBM SPSS Statistics version 23.0 software (IBM, Armonk, NY, USA) [46] will be used for quantitative analysis, and the data analyst will be masked to the groups to minimise bias. The statistical analysis will test the null hypothesis of the non-inferiority of iCBT compared with F2F clinical care between baseline and post-intervention.

#### Non-inferiority margin

A fundamental principle in the analysis of non-inferiority trials is establishing the non-inferiority margin for analysis [45]. To our knowledge, there are no non-inferiority trials using the TFI as the primary assessment measure. Because there is no established margin, this was set using both statistical reasoning and clinical judgment. When developing the TFI, the authors reported that a 13-point difference was considered a clinically significant change in score [23]. Further studies using the TFI have reported larger differences. Fackrell et al. [47], for instance, suggested 22.4 points to be a significant change in score. Using clinical judgment, differences greater than 13 points would not be classed as clinically non-significant. A 13-point non-inferiority margin was judged to be the most reasonable both statistically and clinically.

#### Sample size

The SampSize app using a non-inferiority parallel group assisted with sample size calculations [48]. The α was set to 0.025, power at 90%, and the non-inferiority margin to 13 points with a slightly larger SD of 17 points. The minimal sample size for each group is 39 participants. An additional seven participants will be assigned to each group to account for possible dropouts, estimated on the basis of previous trials of a similar nature to be between 10% and 20% [17, 18]. Therefore, 46 participants will be recruited in each arm.

#### Group differences

Baseline group differences will be analysed using independent samples *t* tests for continuous variables and chi-square tests for categorical variables. In accordance with the recommendations for non-inferiority trials [45, 49], analysis of the primary assessment measure will follow a per-protocol analysis. Participants will be analysed on a per-protocol basis if they complete the post-intervention assessment measures. In addition, per-protocol results will be compared with those using an intention-to-treat paradigm. To enable intention-to-treat analysis, missing value analysis will be done, including Little’s missing completely at random test [50] to test for the missing completely at random assumption. If appropriate, missing data will be imputed through the multiple imputation procedures offered by IBM SPSS Statistics software using the Markov chain Monte Carlo method with five imputation runs [51]. All baseline assessment measure results will be used as predictors.

To determine whether iCBT is at least as effective as F2F care, a confidence interval approach will be used. Non-inferiority of iCBT compared with F2F care will be established if the lower limit of the two-sided 95% confidence interval of the mean difference between these two interventions is less than the non-inferiority margin of 13 points. Owing to the non-inferiority trial, one-sided investigations with a significance of 0.025 will be used. Regression analysis will be used to determine if there is a relationship between baseline and outcome variables in an attempt to identify predictor variables.

#### Qualitative analysis

The open-ended questions in the screening and the semi-structured telephone conversations will be analysed using NVivo 10 software (QSR International, Melbourne, Australia) [52]. The theoretical framework for qualitative analysis will be qualitative content analysis [53].

The study results will be shared in peer-reviewed publications by the present authors and presented at research conferences. A summary of the findings will be available to study participants as well as to members of tinnitus support and tinnitus charity groups.

### Public-patient partnership

This study is designed to let the general public, clinicians and researchers work together in creating an opportunity for a new scientific and clinical intervention. Involvement of a service public-patient partnership is included in this study. A public-patient partnership has been engaged in this research since the development stage and assisted with functionality testing and evaluation of the developed iCBT intervention. This partnership has had input into the study design and study materials used to ensure they are patient-friendly. This group will also serve as an independent point of contact for participants for impartial advice about the study.

## Discussion

In view of the present health care burden of tinnitus, together with estimates that the incidence of tinnitus may only increase, an innovative Internet-based intervention for tinnitus (iCBT) has been developed. Having an additional intervention available for triage may free clinical appointments for those with the greatest need and provide care for those who have limited access to clinical care for geographical or health-related reasons. The feasibility and efficacy of iCBT in the United Kingdom has been established [21, 22]. What has not been determined, however, is how this intervention compares with that of the usual care for tinnitus (i.e., F2F intervention). A strength of this study is that it builds on previous studies by determining feasibility and efficacy in a controlled and powered manner. A further strength is its randomised design, including outcome measures for both tinnitus and possible comorbidities and a follow-up assessment to establish maintenance effects of both interventions.

Studies in Europe that compared iCBT against F2F group interventions reported similar results, regardless of the format of the intervention provided [17, 18, 41]. This study builds on previous research by using an audiologist instead of a clinical psychologist to present the iCBT intervention. Establishing whether audiological professions are able to run an iCBT intervention using their audiological background in tinnitus is important because this is the group of professionals who treat tinnitus in the United Kingdom. This trial is furthermore unique in that it compares iCBT against individual F2F clinical care, as opposed to a group-based approach used in previous studies. The wide range of assessment measures proposed to fully evaluate the effects of the interventions on both tinnitus severity and its comorbidities is a strength of this study. The information obtained will be valuable to help determine for whom this may be a suitable form of intervention, which is an important goal of this study.

The trial has been carefully designed to maximise participant retention by use of regular contact and completion of assessments online. The intervention itself is also in its third revision as improvements are made in a continual manner following participant suggestions. These improvements are envisioned to improve participant retention. The potential impact of this research is significant in that it may provide more accessible tinnitus management options and reduce the burden on current health care and costs of tinnitus-related services. iCBT may be recommended for certain individuals with tinnitus following their clinical examination.

Limitations of the study include the non-uniform nature of clinical care, although attempts have been made to standardise this care as far as possible. Potential barriers may be low recruitment into the study owing to a preference for F2F interventions and lower acceptance of computerised interventions [54]. Publicity regarding the previous research by both the researchers and those who have undertaken the intervention will help improve recruitment. Involvement of the public-patient partnership may also help improve recruitment. A further barrier may be that not all participants will have Internet access. Although 87.9% of adults in the United Kingdom use the Internet [55], this percentage decreases to 38.7% of adults aged 75 years or older. A limitation of the study design is that it is not possible to mask the researcher and participants during the intervention, so they will know in which group they have been placed. Bias will be minimised, however, by masking during randomisation and data analysis.

If iCBT is shown to be an effective addition to the usual tinnitus care in the management of tinnitus, further research will be needed to determine the actual potential of iCBT as a viable intervention. The research will also be required to determine under which circumstances iCBT is effective. Determining participants’ perceptions and experiences of both interventions, as well as what may influence these factors, should also be the focus of future studies. Results of this study are expected to be available during 2018.

### Trial status

Trial recruitment started in October 2016 and is estimated to last until April 2017 (protocol version 6, dated 23 March 2017).

## Additional file

Additional file 1: SPIRIT 2013 checklist: recommended items to address in a clinical trial protocol and related documents. (DOC 122 kb)

## Abbreviations

CBT: Cognitive behavioural therapy
CONSORT: Consolidated Standards of Reporting Trials
ENT: Ear, nose and throat
F2F: Face-to-face
GP: General practitioner
iCBT: Internet-based cognitive behavioural therapy
NHS: National Health Service
SPIRIT: Standard Protocol Items: Recommendations for Interventional Trials
THI: Tinnitus Handicap Inventory
THI-s: Tinnitus Handicap Inventory-Screening Version
TFI: Tinnitus Functional Index
